# Facilitating Tumor Functional Assessment by Spatially Relating 3D Tumor Histology and *In Vivo* MRI: Image Registration Approach

**DOI:** 10.1371/journal.pone.0022835

**Published:** 2011-08-29

**Authors:** Lejla Alic, Joost C. Haeck, Karin Bol, Stefan Klein, Sandra T. van Tiel, Piotr A. Wielepolski, Marion de Jong, Wiro J. Niessen, Monique Bernsen, Jifke F. Veenland

**Affiliations:** 1 Biomedical Imaging Group Rotterdam, Department of Radiology and Medical Informatics, Erasmus Medical Center, Rotterdam, The Netherlands; 2 Department of Radiology, Erasmus Medical Center, Rotterdam, The Netherlands; 3 Department of Nuclear Medicine, Erasmus Medical Center, Rotterdam, The Netherlands; 4 Imaging Science and Technology, Faculty of Applied Sciences, Delft University of Technology, Delft, The Netherlands; Universidade de São Paulo, Brazil

## Abstract

**Background:**

Magnetic resonance imaging (MRI), together with histology, is widely used to diagnose and to monitor treatment in oncology. Spatial correspondence between these modalities provides information about the ability of MRI to characterize cancerous tissue. However, registration is complicated by deformations during pathological processing, and differences in scale and information content.

**Methodology/Principal Findings:**

This study proposes a methodology for establishing an accurate 3D relation between histological sections and high resolution in vivo MRI tumor data. The key features of the methodology are: 1) standardized acquisition and processing, 2) use of an intermediate ex vivo MRI, 3) use of a reference cutting plane, 4) dense histological sampling, 5) elastic registration, and 6) use of complete 3D data sets. Five rat pancreatic tumors imaged by T2*-w MRI were used to evaluate the proposed methodology. The registration accuracy was assessed by root mean squared (RMS) distances between manually annotated landmark points in both modalities. After elastic registration the average RMS distance decreased from 1.4 to 0.7 mm. The intermediate ex vivo MRI and the reference cutting plane shared by all three 3D images (in vivo MRI, ex vivo MRI, and 3D histology data) were found to be crucial for the accurate co-registration between the 3D histological data set and *in vivo* MRI. The MR intensity in necrotic regions, as manually annotated in 3D histology, was significantly different from other histologically confirmed regions (i.e., viable and hemorrhagic). However, the viable and the hemorrhagic regions showed a large overlap in T2^*^-w MRI signal intensity.

**Conclusions:**

The established 3D correspondence between tumor histology and in vivo MRI enables extraction of MRI characteristics for histologically confirmed regions. The proposed methodology allows the creation of a tumor database of spatially registered multi-spectral MR images and multi-stained 3D histology.

## Introduction

Recognizing the impact of the tumor microenvironment on oncogenic processes [1] led to the awareness that successful cancer management involves not only the tumor cells, but also needs to target the tumor microenvironment itself. Therefore, understanding and quantification of the complex molecular and cellular interactions in cancer tissue is of paramount importance. Hence, the imaging of local tumor properties is becoming increasingly important to diagnose, monitor and predict tumor treatment [2], [3]. Magnetic resonance imaging (MRI) has considerable potential in non-invasive tumor characterization, as a multitude of scanning techniques can be employed. However, the exact relation between the signal intensities in MRI and the underlying pathophysiology is not always understood. Thorough understanding of the MRI oncogenic signatures involves an accurate spatial correlation of MRI and histology, offering a means to verify MRI findings. On the other hand, to create histological images the tumor tissue undergoes excision, fixation by formalin followed by dehydration, paraffin embedding, sectioning, and rehydratation during staining. An important side effect of this process is the significant tissue deformation which inevitably changes the tumor appearance. This severely complicates the registration of *in vivo* MRI to histological sections. Besides the loss of the tumor 3D integrity, the registration is also complicated by the inherent differences in image characteristics between color histological images and gray scale MRI images.

Although the field of multi-modality registration has evolved considerably, the literature specifically dealing with registration of MRI to histology is limited, especially for *in vivo* MRI acquisitions. The first attempts to register histology and MRI were part of an effort to establish brain atlases, starting with affine registration [4] and advancing to piece-wise affine models [5]. Although affine registration achieved good initial results in these applications, they are inadequate to deal with non-linear distortions that occur during tissue excision and histological processing. Elastic registration for linking MRI with histology using surface matching has also been considered [6], [7]. Unfortunately, the reported results are limited to global matching of MRI volumes. Other studies [8] included point-based registration using manually placed landmarks. Besides being time consuming, these studies are also prone to intraobserver variability due to involvement of human interaction.

In oncological applications, co-localization of histology and MRI is often based on simple visual evaluation of local tissue features [9] and is therefore subjective and limited to a small number of histological sections. To facilitate rigid alignment several fiducial marker systems have been introduced [10], [11], [12]. These markers are physical implants that are clearly visible in all imaging modalities. Even though they might be useful for animal imaging, the use of fiducial markers in clinical applications is rarely possible. Therefore, as an alternative, distinctive image features (within or at the surface) of the object under registration can be used to facilitate image alignment. For example, *in vivo* MRI of whole rat brain [13] and human prostate [14], [15] was related to their histological sections by point-based registration using manually placed [13], [15] or automatically established [14] landmark points. Although these internal landmarks have successfully assisted the registration of a complete organ, this compromises the registration accuracy within the tumor as it registers the organ instead of the tumor. Even though these methods solve part of the registration problem by using block-face images, they fail to account for 3D deformation as they use a limited number of histological sections.

To overcome the limitations of these methods, we propose the registration of complete 3D histology with *in vivo* MR images of the tumor tissue, i.e. excluding surrounding tissue. The aim of this work is to develop a methodology for establishing an accurate 3D relation between high resolution *in vivo* MRI and corresponding 3D histology of tumor tissue. The key features of the methodology are: a standardized imaging and histology method, acquisition of an intermediate *ex vivo* MRI, use of a reference cutting plane, a dense histological sampling, elastic (B-spline) registration, and use of the complete 3D data set.

## Materials and Methods

**Figure 1:**
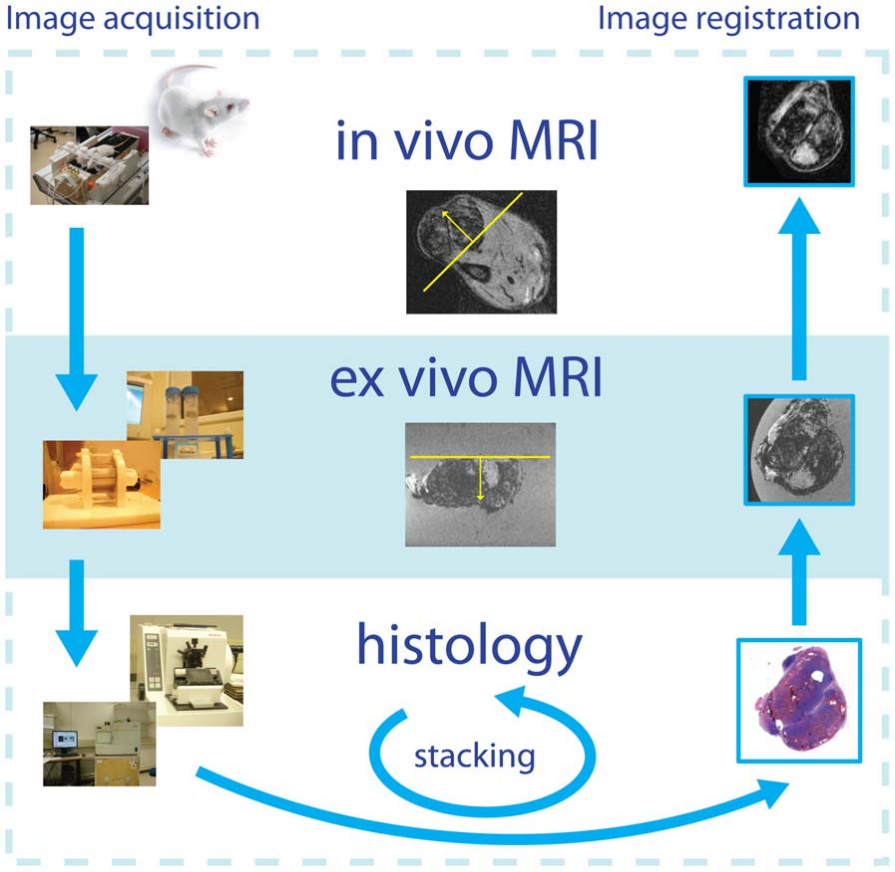
Overview of the processing steps (left-hand side) and the image registration and stacking procedures (right-hand side). To facilitate the registration of *in vivo*, *ex vivo* and histology images, the tumor orientation was tracked by color coding the different tumor surfaces and by creating a reference cutting plane. This reference plane was created by slicing off a thin section of the whole tumor volume along the longest tumor axis subsequent to fixation. Although the reference plane is not physically present in *in vivo* MRI, the knowledge of its orientation is crucial to perform image resampling prior to registering *in vivo* MRI with *ex vivo* MRI [16].

**Figure 2:**
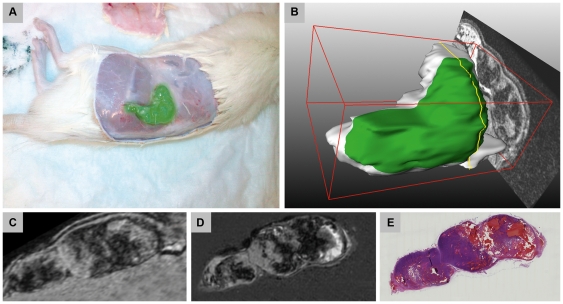
Illustration of subcutaneous tumor position. Tumor at onset of dissection (**A**) and as a 3D in vivo MRI tumor volume rendering (**B**), the subcutaneous side of the tumor is marked in green. A yellow line represents the cutting plane orientation along the longest tumor axis and perpendicular to the subcutaneous tumor side. The second row images show the corresponding slices of in vivo MRI (**C**), ex vivo MRI (**D**) and as histological section (**E**).

Figure 1 is a schematic overview of the proposed methodology, which consists of a number of image acquisition steps (top-to-bottom) and image registration (bottom-up) steps. To facilitate the registration of *in vivo*, *ex vivo* and histology images, we kept track of the tumor orientation by color coding the different tumor surfaces and by creating a reference cutting plane. This reference plane was created, after fixation, by slicing of a thin section of the whole tumor volume along the longest tumor axis and perpendicular to the subcutaneous side of the tumor. Although the reference plane is not physically present in *in vivo* MRI, the knowledge of its orientation is crucial to perform image resampling prior to registering *in vivo* MRI with *ex vivo* MRI [16], [17]. Figure 2 shows the tumor at onset of dissection, and the location of the reference plane in the volume rendered tumor in MRI.

### Animal and tumor model

For this study, approval from the Ethical Committee of the Erasmus MC was obtained (Erasmus MC OZP 112-08-06). All investigations were carried in accordance with the requirements of the institution concerned, and also conform to the general requirements in the Netherlands regarding animal studies. Five male Lewis rats (Harlan-CPB, Austerlitz, The Netherlands), with a mean body weight of 300 g, were inoculated subcutaneously in the right hind limb with 10^6^ pancreatic (CA20948) tumor cells suspended in Hanks' balanced salt solution. The inoculated pancreatic tumors grow just beneath the skin as an encapsulated mass on top of the muscle tissue, with a preferred growth direction parallel to the skin (see Figure 2-A). The tumor boundaries are well defined and the tumor is easy to separate from surrounding tissue. The animals were inspected daily for tumor growth and general appearance. The tumors were imaged using MRI when they reached approximately 10 mm in diameter. Before MRI, the animals were anesthetized by intraperitoneal injection of medetomidine (Sedator, Eurovet Animal Health B.V., Bladel, The Netherlands) and sufentanil (Sufenta forte, Janssen-Cilag B.V., Tilburg, The Netherlands). During the imaging, the animals were kept at a temperature of 38–39°C by warm water mattresses. After *in vivo* MRI, animals were euthanized, and the complete undamaged tumors were dissected. During the dissection, the tumor surfaces were dyed to track the in vivo tumor orientation by marking the subcutaneous, the head, the tail and dorsal side of the tumor. Figure 2-A shows the subcutaneous tumor position at onset of dissection. Immediately after dissection, tumors were placed in 200 ml 10% buffered formalin (Boom, The Netherlands). A crucial step to facilitate alignment between in vivo MRI, and 3D histology stack is the knowledge of tumor orientation in all imaging modalities concerned [16], [17]. We created a reference plane by slicing of a thin section of the whole tumor volume along the longest tumor axis and perpendicular to the subcutaneous side of the tumor. The reference plane is illustrated in Figure 2-B as a yellow line. The tumors were washed first to avoid possible T2* artifacts due to remaining formalin concentrated on the tumor surface. Washing the tumors was achieved by sinking them into saline solution and drying the remaining moisture by paper towels. Subsequently, tumors were suspended in 1% agar dissolved in phosphate buffered saline (PBS, AbD Serotec, MorphoSys, Munich, Germany) to facilitate ex vivo MRI acquisition by restricting tissue motion and air-tissue MRI artifacts.

### Magnetic resonance imaging

For the in vivo MRI acquisition parameters were: TR/TE = 23.2/8.9 ms, flip angle of 10°, field-of-view (FOV) of 50×50 mm^2^, image acquisition matrix of 320×256 with a slice thickness of 0.4 mm (acquired voxel resolution = 0.156×0.195×0.4 mm^3^) and a resampled matrix of 512×512 using zero-filling for a reconstructed voxel size of 0.098×0.098×0.2 mm^3^. For the ex vivo MRI acquisition parameters were: TR/TE = 42.2/20.9 ms, flip angle of 15°, field-of-view (FOV) of 50×50 mm^2^, image acquisition matrix of 320×256 with a slice thickness of 0.4 mm (acquired voxel resolution = 0.094×0.118×0.4 mm^3^) and a resampled matrix of 512×512 using zero-filling for a reconstructed voxel size of 0.059×0.059×0.2 mm^3^. For both in vivo and ex vivo MRI read bandwidth was 48.8 Hz/voxel, no flow compensation or saturation pulse, two averages, frequency encoding = left-right, and the phase encoding direction = up-down. The total acquisition time was less than 20 minutes for both *in vivo* and *ex vivo* acquisitions. No acceleration was used for imaging.

### Histolological processing

Following the *ex vivo* MR imaging, tumors were processed in a Histokinette, and subsequently embedded in paraffin. The histological data consisted of 4-µm thick sections (cut from the reference plane onwards, see Figure 1 and Figure 2) mounted on glass slides, and stained with hematoxylin and eosin (H&E). Depending on the tumor size, up to 40 sections (4-µm thickness each) were mounted at intervals of 80 µm. The procedure also enables to acquire histological sections with different stains. The slides were digitized using the NanoZoomer Digital Pathology (C9600, Hamamatsu, Japan) at 20× magnification, which resulted in a pixel size of 3.64 µm.

### Registration

We first provide an outline of the different parts in the automatic registration procedure which were performed using Elastix [18]. The details of the image registration are included in Text S1 [19], [20], [21], [22], [23], [24], [25], [26], [27] with the basic components of the registration framework are illustrated in Figure S1. Between the different image acquisition steps (Figure 1) a tumor undergoes deformations with respect to its original *in vivo* shape. As these deformations differ in nature and scale, the registration procedure consists of three distinct parts. All registrations use contrast in image intensities to perform the registration automatically.

1. Reconstruction of tumor 3D histology by rigid registration of digitized adjacent H&E sections and adjustment of the slice thickness, referred to as stacking.
2. Volumetric alignment of 3D histology stack and 3D *ex vivo* MRI using a three-step strategy (rigid, affine, and elastic registration), referred to as stack2ex.
3. Volumetric alignment of 3D *ex vivo* MRI to 3D *in vivo* MRI using a three-step strategy (rigid, affine, and elastic registration), referred to as ex2in.

All separate registrations were performed using Elastix [18]. To achieve the desired volumetric alignment of 3D histology to *in vivo* MRI, the separate transformations (the results from stack2ex and ex2in registrations) were concatenated automatically. The final concatenated geometric transformation, referred to as stack2in, was applied to the 3D color histology stack which aligns it to the *in vivo* MRI.

#### Stacking

**Figure 3:**
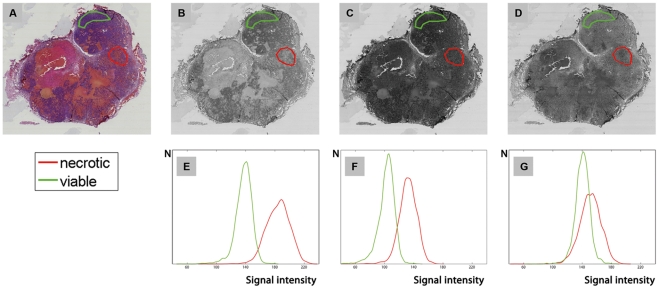
The distribution of separated image channels from a H&E section. We used the red image channel to perform the registration as it provides the best separation between signal intensities of necrotic and viable volumes of interest (VOIs) and presumably the best image contrast. A H&E stained histological section (**A**) and three separate color channels, red-green-blue, (**B–D**) with corresponding histogram distributions of the vital (green) and necrotic (red) tumor regions (**E–G**).

As the first step in the automatic registration process, we automatically reconstructed 3D histological volume by rigid registration of adjacent H&E stained images. To optimally exploit the digital image information, considering the necrotic and viable tissue, the information content of separate image channels was evaluated. We used the red image channel in the registration as it provides the best separation between signal intensities of necrotic and viable volumes of interest (VOIs) and presumably the best image contrast (Figure 3 presents histograms of these VOIs). The series of 2D histological slices (red channel) were reconstructed iteratively into a 3D volumetric image. The resulting transformations were applied to the other two (green and blue) image channels, resulting in a 3D color histology stack. Subsequently, the slice thickness was set to 80 µm, i.e. the physical distance between subsequent sections.

#### stack2ex

The second automatic registration step, aligning the 3D histology with *ex vivo* MRI, is greatly facilitated by the definition of the reference cutting plane (see Figure 2), i.e. both images (*ex vivo* MRI and histology stack) start at the same position (reference plane). This provides the initialization for a three-step registration strategy of gradually increasing degrees of freedom, starting as rigid registration, followed by affine registration, and finalized by elastic refinement.

#### ex2in

Prior to the third automatic registration step, the knowledge of the reference plane within *in vivo* 3D-T2^*^w MRI (see Figure 1) was used to realign and resample the *in vivo* data according to the *ex vivo* MRI orientation. This ensures similar orientation and rough alignment of *in vivo* MRI and *ex vivo* MRI. First, rigid registration was performed, followed by affine transformation allowing isotropic scaling to account for volume changes, and finalized by elastic registration.

### Evaluation

#### Evaluation of registration accuracy

The resulting alignment of *in vivo* 3D-T2^*^w MRI with 3D histology stack was qualitatively evaluated by two observers using visual inspection with a moving quadrant view, and quantitatively evaluated using anatomical landmarks (e.g., characteristic features in the tumor and on the contour). For the quantitative evaluation, ten clearly identifiable anatomical landmarks were initially defined on the color 3D histology stack. Subsequently, two observers independently annotated the corresponding anatomical landmarks in the *in vivo* 3D-T2*w MRI. To evaluate registration accuracy, the root mean squared (RMS) distance between the corresponding points in the *in vivo* MRI and 3D histology was calculated before registration, and after the two registration steps (i.e., rigid and elastic). Furthermore, the interobserver variability was estimated by computing the RMS distance between the corresponding points of the two observers on the MRI.

#### Evaluation of reference plane

The reference plane greatly facilitates the registration procedure. The difference in reference plane position between two 3D images after registration, measures the initial reference plane error. To quantify the error in reference plane positioning, the out-of-plane angulation is estimated as the rotation component of the rigid registration for both steps (stack2ex and ex2in).

#### Tumor volume change

Tumor global volume change between *in vivo* MRI, *ex vivo* MRI and histology was established by computing the determinant of the corresponding affine transformation for both registration steps (stack2ex and ex2in). The tumor local volume change for the different histological regions was also estimated. For this purpose, three volumes of interest (VOIs) representing viable, necrotic and hemorrhagic regions were delineated in the color 3D histology stack. This provides three masks which were warped using the transformation, provided by the corresponding registration step, to match the *in vivo* MRI. For each region (viable, necrotic and hemorrhagic) and both registration steps (stack2ex and ex2in), the change in volume was estimated before and after registration: see Eq. (1).
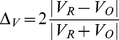
(1)where V_O_, and V_R_ represent the VOIs before and after registration, respectively.

#### Facilitating MRI characteristics identification

To identify image characteristics of *in vivo* 3D-T2^*^w MRI, histograms of histologically confirmed VOIs were used to estimate the probability density function (pdf). For each VOI's histogram, the pdf interquartile range was then used for automatic segmentation of the *in vivo* 3D-T2^*^w MRI.

## Results

### Evaluation of registration accuracy

**Figure 4:**
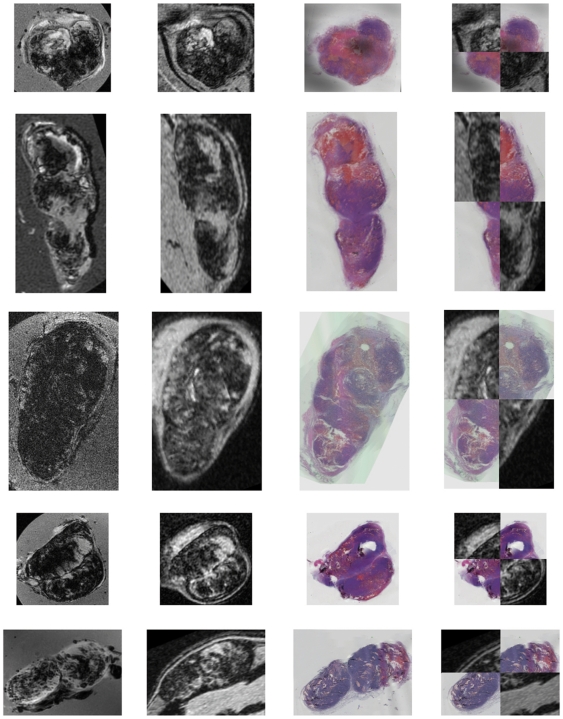
Final registration results for five tumors. Registered *ex vivo* T2*-w MRI (first column), *in vivo* T2*-w MRI (second column), registered color 3D histology (third column), and checkerboard view of *in vivo* and registered histology (fourth column).

Figure 4 shows the results of the separate registration steps (stack2ex and ex2in) and the concatenation of those registrations (stack2in) for the five tumors. The checker board view (Figure 4; fourth column) of the registered *in vivo* 3D-T2^*^w MRI and the 3D histology shows that good alignment has been achieved. For all five tumors, the final registration (stack2in) was evaluated as excellent for 25% and good for 53% of the registered slices. For 13% of the slices the registration was evaluated as fair, for the remaining 9% as poor. The registration of the slices towards the tumor borders was in general less accurate than the registration of central slices.

Table 1 presents the RMS distance error for 10 landmark positions averaged over all five tumors after final registration (stack2in). By utilization of the reference plane, the initial average accuracy was already 1.4 mm. After registration, the average accuracy increased from 1.4 mm to 0.7 mm. When compared with the *in vivo* pixel size, the average accuracy increased from 15 to 7 pixels. The final accuracy of 0.7 mm corresponds on average with 30–50 cells. To assess the uncertainty of the manual annotations, we computed the interobserver variation, which was in the order of 0.7 to 0.9 mm.

**Table 1:** Average root mean squared distances (mm) for the different registration steps, averaged over all 5 subjects.

	Observer 1	Observer 2	Average	Inter-observer
**Initial**	1.2±0.6	1.6±0.7	1.4±0.6	0.9±0.6
**Rigid**	1.1±0.5	1.0±0.4	1.0±0.4	0.7±0.3
**Elastic**	0.8±0.3	0.6±0.2	±0.3	0.7±0.4

### Evaluation of reference plane

Error in the reference plane positioning, measuring the remaining 3D mismatch, was established for both registration steps separately. Table 2 summarizes the angulation as averaged over all five subjects. The absolute angulation for st2ex registration was 1.4±1.30%, ranging from −2.52 to 3.08, and for ex2in registration was 2.3±1.34% with a range of −2.79 to 4.00. This shows that directional mismatch between the resampled *in vivo* MRI and *ex vivo* MRI, and between *ex vivo* MRI and histological sections were minimal.

**Table 2:** Summarized registration results averaged over all five subjects.

Registration	ΔV (global)	Angulation	ΔV (necrotic)	ΔV (viable)	ΔV (hemorrhagic)
step	[%]	[°]	[%]	[%]	[%]
**stack2ex**	−1.9±0.07	1.4±1.30	12.2±6.4	11.9±6.4	11.0±8.7
**ex2in**	13.2±0.05	2.3±1.34	16.9±6.8	16.0±4.4	11.2±15.0

### Tumor volume change

On average, the global tumor volume expanded 1.9% after sectioning. The same specimens shrank on average 13.2% after chemical fixation. Table 2 summarizes global and local volume change (per VOI) averaged over all five subjects. All histologically different regions (i.e., viable, necrotic, and hemorrhagic) expanded similarly after sectioning. On the other hand, we observed a significant difference in deformation between different histologically confirmed regions. That is, the shrinkage after chemical fixation is different for the hemorrhagic region compared with the necrotic and viable regions.

### Facilitating MRI characteristics identification

**Figure 5:**
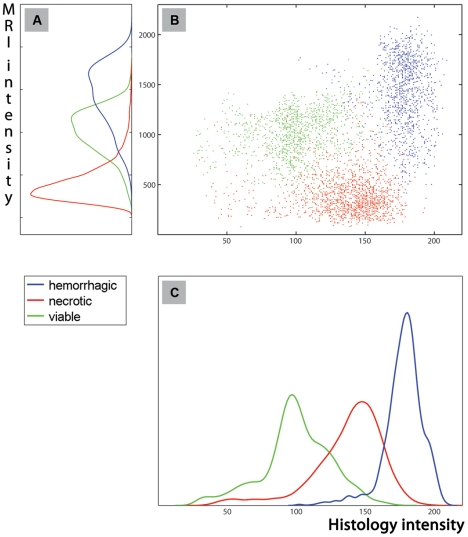
The illustration of signal intensity correspondence between *in vivo* T2*-W MRI and registered 3D histology for three VOIs (e.g., necrotic-red, viable-green, and hemorrhagic-blue). The 3D correspondence of tumor histology and *in vivo* MRI enables extraction of MRI characteristics for histologically defined regions. This is illustrated in scatter plot (**B**) and using the histogram-based probability density function of the registered histology (**C**) which clearly separated the different tissue types in the H&E stained images. The corresponding probability density function of *in vivo* 3D-T2^*^w MRI (**A**) demonstrates that viable and hemorrhagic regions cannot be separated using solely *in vivo* 3D-T2^*^w MRI signal intensities. Nevertheless, necrotic regions can be effectively separated from the other two histologically confirmed regions.

**Figure 6:**
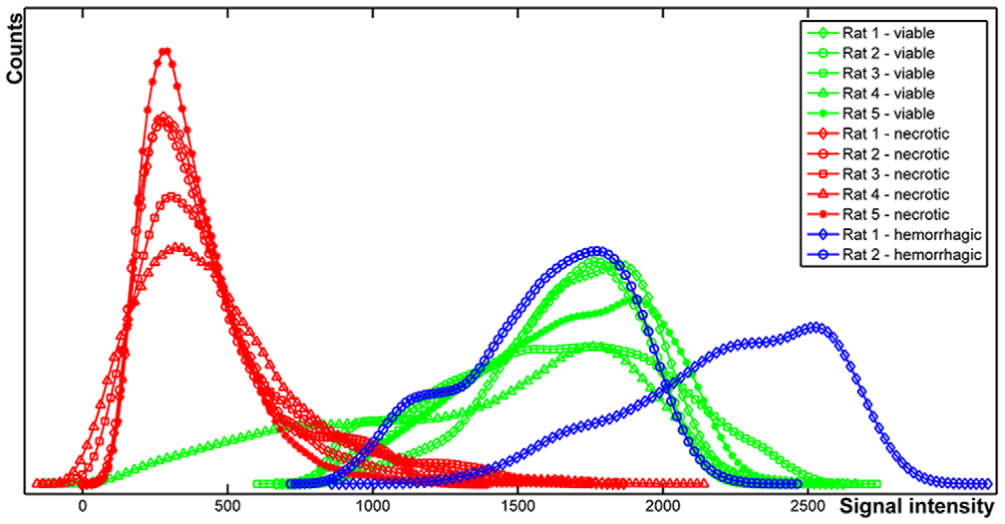
The group-wise probability density functions distributions of *in vivo* 3D-T2^*^-w MRI. Three VOIs (e.g., necrotic-red, viable-green, and hemorrhagic-blue) were annotated in histological sections and used for segmentation of automatically aligned *in vivo* MRI. The excessive hemorrhagic regions are visible in two out of five subjects. It demonstrates the similar gray value ranges for each VOI. When considering the probability density function for all tumors, the necrotic regions were significantly different from other histologically confirmed regions (i.e., viable and hemorrhagic). However, the viable and the hemorrhagic regions showed a large overlap in T2^*^-w MRI signal intensity.

The 3D correspondence of tumor histology and *in vivo* MRI enables extraction of MRI characteristics for histologically defined regions. This is illustrated using the histogram-based pdf of the registered histology (Figure 5-C) which clearly separated the different tissue types in the H&E stained images. The corresponding pdf of *in vivo* 3D-T2^*^w MRI (Figure 5-A) demonstrates that viable and hemorrhagic regions cannot be separated using solely *in vivo* 3D-T2^*^w MRI signal intensities. Nevertheless, necrotic regions can be effectively separated from the other two histologically confirmed regions. Figure 6 evaluates the pdf of *in vivo* 3D-T2^*^-w MRI for all subjects demonstrating similar gray value ranges for each VOI. When considering the pdf for all tumors, the necrotic regions were significantly different from other histologically confirmed regions (i.e., viable and hemorrhagic). However, the viable and the hemorrhagic regions showed a large overlap in T2^*^-w MRI signal intensity.

**Figure 7:**
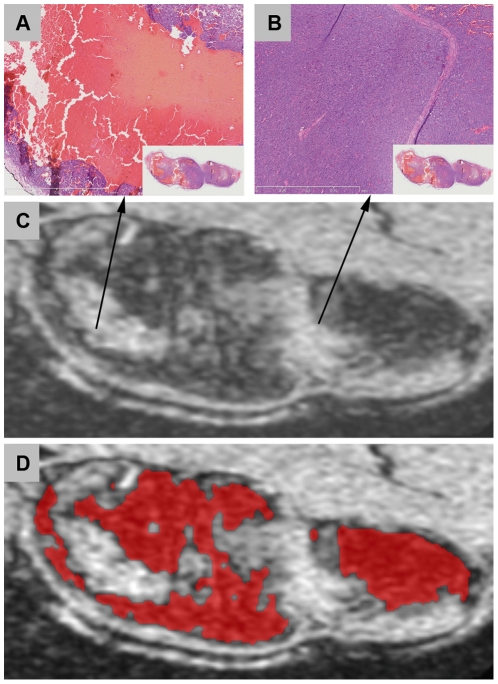
Details from a H&E stained section and its corresponding MRI slice. Histological section (**A–B**) shows the difference in histological appearance, whereas the MRI appearance in 3D T2*-w MRI (**C**) is similar. The necrotic segmentation, superimposed on 3D T2*-w, is shown in red (**D**).

To confirm the findings shown in Figure 5 we used the histogram-based pdf to define the interquartile intensity ranges for histologically confirmed regions. These ranges were used for automatic segmentation of *in vivo* 3D-T2^*^w MRI signal intensities. Figure 7 illustrates the necrotic segmentation superimposed on 3D T2*-w MRI. The viable and hemorrhagic tissues cannot be separated based on the T2*-w MRI intensity.

## Discussion

This proposed methodology, i.e. aligning histological tissues sections to *in vivo* MRI, consists of a number of image acquisition and image registration steps that have been evaluated. The methodology is assembled around two separate registration steps, both exploiting a three-step strategy of gradually increasing degrees of freedom (rigid, affine, and elastic transformation), which allow for a coarse-to-fine scheme. To enable the registrations, we kept track of the tumor orientation by color coding the different tumor surfaces and by creating a reference plane. Qualitative and quantitative evaluation of the registration and protocol accuracy was performed.

During the registration evaluation, the alignment of tumor surface and internal structures was qualitatively evaluated as accurate. Quantitatively, we achieved an average accuracy of 0.7 mm after the registration. The results involving two observers to estimate the RMS error showed similar trends in increasing accuracy with increasing degrees of freedom. The interobserver variation of the manual annotation was approximately 0.7 mm. This is an indication of the limitation of the measurement method; smaller distances could not reliably be measured. The RMS distance after elastic registration is of the same order. Evaluation of the protocol accuracy shows that a 3D-registration method complemented by standardized acquisition is essential to accurately align histology to *in vivo* MRI. Excision and fixation of the tumor resulted in an average shrinkage of 13%. However, the sectioning of the tumor enlarged the tissue by 1.9%.

Park et al. registered prostates imaged using *in vivo* MRI, *ex vivo* MRI after prostatectomy, block-face photographs, and histological sections [15]. They used block-face photographs to reconstruct the original histology. Registration was performed by point-based registration using manually placed landmarks. They moved towards 3D registration using three consecutive slices during histology-to-MRI registration. Although studies have registered whole-prostate histology to *in vivo* MRI, to our knowledge the present study is the first attempt to register pancreatic tumors. Our methodology intentionally excludes the use of block-face images as this would complicate image acquisition and registration when acquiring large number of histological sections. Compared to the method proposed by Park et al. [15] our method uses denser histological sampling, no user interaction is required during the registration procedure, and the whole image content of the tumor volume is utilized for registration.

This study presents the successful development and careful evaluation of a combined methodology for alignment of tumor histological sections to *in vivo* MRI. At the same time, it demonstrates the importance of integrated methodology between imaging and registration. The established 3D correspondence between tumor histology and *in vivo* MRI enables extraction of MRI characteristics for histologically confirmed regions. We showed that, based on T2^*^-w MRI signal intensity, automatic identification of necrotic tissue is feasible. However, based on T2*-w MRI, the separation of hemorrhagic and viable tissue was not possible. The hypo-intense areas in T2*-w MRI seem to correspond to necrotic tissue, see Figure 7. However, this conclusion should be taken cautiously as deoxyhemoglobin and hemosiderin can also cause low intensity on T2*-w MRI [28]. As those may be undistinguishable in the T2*-w MRI, tumor necrosis may have been overestimated by MRI analysis.

**Figure 8:**
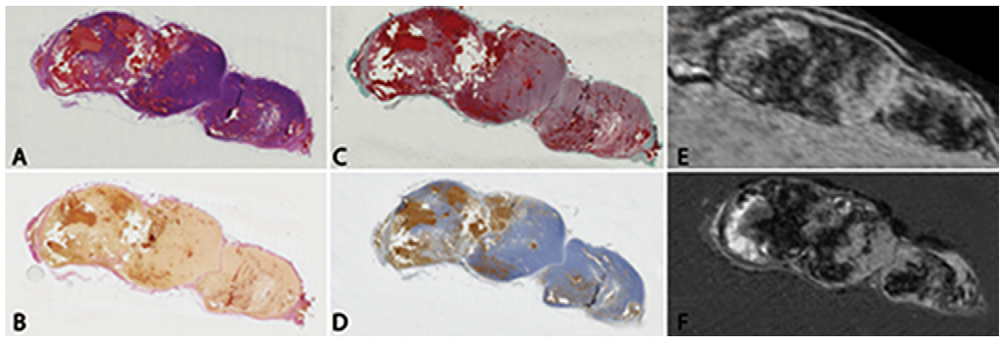
Multi-stained histology dataset. H&E (**A**), Goldner (**B**), van Gieson (**C**) and Peroxidase (**D**) stained consecutive sections with the corresponding *in vivo* MRI (**E**) and intermediate *ex vivo* MRI (**F**). The slice thickness of all histology sections is 4 µm, and the distance between the consecutive histology sections (**A–D**) is 8 µm.

This work is a first step in MRI tumor characterization. When the basic correspondence between *in vivo* MRI and 3D H&E histology can be established, the extension to multi-spectral MR images and multi-stained histological sections is a logical next step. Different histological stains highlight different aspects of the tumor, in Figure 8 the spatial correspondence between the *in vivo* MRI, the *ex vivo* MRI and multi-stained histological sections is shown. This work can be used to create a database consisting of multi-spectral MRI images and multi-stained 3D reconstructed histology that may be an essential and valuable source for understanding MR images, and highly beneficial in the process of identifying MRI tumor characteristics.

Some modifications are envisioned which need exploring, as they will increase the robustness and accuracy of the technique without significantly increasing processing time. In the protocol used, the hyper-intense regions cannot be specified based on solely T2*-w MRI as shown in Figure 7 and Figure 8. The use of multi-modality MRI images is expected to enable a more detailed differentiation between tissue types by combining the different contrast mechanisms present in the MRI sequences. For example, contrast enhanced (CE) MRA or DWI-MRI may create a contrast between vital and hemorrhagic regions. Multi-modality MRI images will therefore refine the registration and offer a more detailed biological profile of the tumor.

## Supporting Information

Figure S1 **The basic components of the registration framework containing two input images, a transform, a metric, an interpolator and an optimizer (adopted from Ibanez et al.** [**21**] **).**
(TIF)

Text S1 **Registration summary.**
(DOC)
