# Supplementary figures and images for: Facilitating Tumor Functional Assessment by Spatially Relating 3D Tumor Histology and In Vivo MRI: Image Registration Approach
Source: PLoS One. 2011 Aug 29;6(8):e22835. doi: 10.1371/journal.pone.0022835 (PMC3163576; doi:10.1371/journal.pone.0022835)

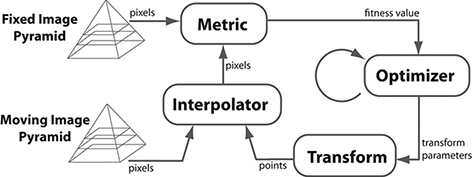

Supplement: Figure S1 — The basic components of the registration framework containing two input images, a transform, a metric, an interpolator and an optimizer (adopted from Ibanez et al. [21] ). (TIF) [file pone.0022835.s001.tif]
