# Supplementary material for: Facilitating Tumor Functional Assessment by Spatially Relating 3D Tumor Histology and In Vivo MRI: Image Registration Approach
Source: PLoS One. 2011 Aug 29;6(8):e22835. doi: 10.1371/journal.pone.0022835 (PMC3163576; doi:10.1371/journal.pone.0022835)
Supplement: Text S1 — Registration summary. (DOC) [file pone.0022835.s002.doc]

**Registration summary**

Medical image registration (also referred to as fusion, superposition, matching or alignment) considers the process of transforming different data sets into one coordinate system to achieve biological, anatomical or functional correspondence. This transformation is necessary to compare (or integrate) the information from different modalities, from baseline to follow-up scans, from pre to post contrast scans, from pre to post treatment scans, and between different subjects. Thereby, it reveals additional information not apparent in the separate images. A comprehensive review on the subject is given by . The choices regarding implementation of the different registration components can have considerable impact on the registration results. All registrations in this paper were performed using Elastix , a publically available software for medical image registration based on Insight Segmentation and Registration Toolkit (ITK) . The basic components of the registration framework are illustrated in Figure S2 and are described in more detail in this section.

One image, called the *moving image I_M_*, is deformed to fit the other image, called the *fixed image I_F_*. More formally, registration is the problem of finding a *coordinate transformation* ***T*** that aligns *I_M_*(***T***) spatially with *I_F_*. The quality of alignment is defined by a cost function *C*(***T*** ; *I_F_ , I_M_*). The optimal coordinate transformation is estimated by minimizing the cost function with respect to ***T***, usually by means of an iterative optimization method embedded in a hierarchical (multi-resolution) scheme.

Mathematically, the registration is defined as an optimization problem where the cost function *C* is minimized with respect to ***T***, eq. S1.

$\mu^{\wedge} =\arg\min_{\mu} C(T_{\mu};I_{F},I_{M})$ Eq.S1

where the subscript ***µ*** indicates the transform parameterization, and contains the transformation parameters. The minimization problem (eq. S1) is solved with an iterative optimization method, usually in a multi-resolution setting.

**Cost Function.** The cost function *C* measures the similarity between the images. We have used mutual information (MI) as it only assumes a statistical relation between image intensities, and is therefore suitable for registration of images acquired by different modalities . The MI was parameterized using a 32-bin image intensity histogram.

**Optimizer.** To optimize the metric criterion with respect to the transform parameters, an iterative optimization procedure is employed. We used an adaptive stochastic gradient descent algorithm .

**Transform.** For the coordinate transformation ***T***, different deformations with varying degrees of freedom are available. In order of increasing transformation flexibility: the rigid, the affine, the deformable (non-rigid, or elastic) transformations. After proper rough initialization, achieved by reference plane orientation, we used a three-step strategy of gradually increasing degrees of freedom, starting with rigid registration, followed by affine registration, and finalized by non-rigid refinement. The displacement field was parameterized using a third order B-spline model.

**Sampling Strategies.** To compute the cost function *C,* a set of samples needs to be selected. The most straightforward strategy is to use all voxels from the fixed image, which has the obvious disadvantage that it is time consuming for large images. A common methodology is to use a subset of voxels, selected on a uniform grid, or sampled randomly. Another strategy is to pick only those points that are located on striking image features, such as edges. In our application, we used a subset of voxels sampled randomly.

**Interpolation.** For computation of the cost function, the moving image *I_M_* needs to be evaluated at non voxel positions which requires the intensity interpolation. Several interpolation methods (varying in quality and speed) have been proposed and compared by Pluim et al. . In this paper we have used a third order B-spline interpolation.

**Optimization.** Multi-resolution (hierarchical) strategies are an important aspect of image registration . Such coarse-to-fine schemes in general improve registration accuracy and increase robustness by eliminating local minima of the cost function at coarser scales. The basic idea is to perform the first registration at a coarse scale with down sampled images. The spatial mapping determined at the coarse level is then used to initialize registration at the next finer scale. This process is repeated until it reaches the finest scale.

**References**

19. Modersitzki J (2003) Numerical Methods for Image Registration: Oxford University Press.

20. Hill DL, Batchelor PG, Holden M, Hawkes DJ (2001) Medical image registration. Phys Med Biol 46: R1-45.

21. Ibanez L, Schroeder W, Ng L, Cates J (2005) The ITK Software Guide: Kitware, Inc.

22. Rueckert D, Sonoda LI, Hayes C, Hill DL, Leach MO, et al. (1999) Nonrigid registration using free-form deformations: application to breast MR images. IEEE Trans Med Imaging 18: 20.

23. Maes F, Collignon A, Vandermeulen D, Marchal G, Suetens P (1997) Multimodality image registration by maximization of mutual information. IEEE Trans Med Imaging 16: 187-198.

24. Thevenaz P, Unser M (2000) Optimization of mutual information for multiresolution image registration. IEEE Trans Image Process 9: 2083-2099.

25. Klein S, Staring M, Pluim JP (2007) Evaluation of optimization methods for nonrigid medical image registration using mutual information and B-splines. IEEE Trans Image Process 16: 2879-2890.

26. Pluim JPW, Maintz JBA, Viergever MA (2000) Interpolation artefacts in mutual information-based image registration. Computer Vision and Image Understanding 77: 211-232.

27. Lester HA, S.R (1999) A survey of hierarchical non-linear medical image registration. Pattern Recognition 32: 129-150.
